# Factors associated with attrition in a longitudinal study of health risk behaviours and conditions among adolescents in Ibadan, Nigeria

**DOI:** 10.1371/journal.pone.0320150

**Published:** 2025-04-04

**Authors:** Adesola O. Olumide, Emmanuel S. Adebayo, Sharon Fonn

**Affiliations:** 1 Institute of Child Health, College of Medicine, University of Ibadan and University College Hospital, Ibadan, Oyo, Nigeria; 2 Institute of Child Health, College of Medicine, University of Ibadan, Ibadan, Oyo, Nigeria; 3 School of Public Health University of the Witwatersrand, Johannesburg, South Africa; 4 School of Public Health and Community Medicine, University of Gothenburg, Gothenburg, Sweden; PLOS: Public Library of Science, UNITED STATES OF AMERICA

## Abstract

**Background:**

Use of longitudinal design in research improves understanding of adolescent health. In this paper, we present factors associated with attrition in a pilot longitudinal study among adolescents in Ibadan, Nigeria.

**Methods:**

Adolescents were recruited from private and government-owned junior secondary schools using multi-stage sampling and interviewed over three data collection waves (2017, 2018 and 2019).

**Results:**

A total of 1067 (99.4%) of the 1073 adolescents recruited were willing to participate and were interviewed in wave one. Mean age at baseline was 11.9 ± 1.2 years and 34.9% owned a personal mobile phone. Of the 1067 adolescents, 192 (18.0%) were not willing to be followed up while 875 (82.0%) were willing to be followed up by home visit (70.2%), phone call (21.3%), text message (14.3%) or online chat-based message (4.8%). Overall attrition rate (proportion of adolescents lost to follow-up during waves two and/or three compared with the baseline sample) was 66.5% with 396 (37.1%) and 315 (46.9%) respondents lost to follow-up during waves two and three respectively. Common reasons for attrition were use of pseudonyms instead of real names, which many adolescents could not remember during subsequent data collection waves, relocation to a different school or neighborhood, school drop-out and closure of two schools. Adolescents in private versus government-owned schools (AOR = 3.35; CI =  2.39 – 4.69), those who did not have personal mobile phones (AOR = 1.43, CI =  1.03 – 1.98) and those engaging in remunerated work (AOR =  2.04, CI =  1.19 – 3.49) were more likely to be lost to follow-up.

**Conclusions:**

Attrition was high despite high willingness to participate in the study. Whereas technology has made follow-up of study participants in high-income countries easier, multiple, and cost intensive methods to minimize attrition may be required in low-resource settings.

## Introduction

Adolescence is a stage of transition from childhood to adulthood marked by a tendency to experiment and initiate risky behaviors [1–5]. While researchers have studied adolescent behaviours in many locations [5–8] context matters, making research in Nigeria important. Use of the cross-sectional study design in adolescent-focused research is widespread. While this design is appropriate, a longitudinal design has additional advantages such as providing information on incidence and temporality; and the possibility of predicting future outcomes based on earlier identified factors [9]. Examples of longitudinal studies conducted among adolescents include the CogBIAS longitudinal study in the UK that examined psychological development during the adolescent years [10,11] and the ‘Birth-to-Twenty’, study in South Africa [12]. The ‘Birth-to-Twenty’ study examined predictors of sexual risk behaviours, metabolic syndrome, and social marginalization among the adolescent participants who initially belonged to the Birth-to-Ten cohort [12]. Despite the many advantages of longitudinal studies, they also have challenges, notably the cost of recruitment and follow-up, and problems of attrition [9,13,14] Longitudinal studies involving adolescents have reported attrition rates ranging from three percent [15] to as high as 75% [16–21]. Various reasons for attrition including participant and family mobility [21,22]; change of school, change in home address, relocation, or refusal to continue in the study have been identified [20]. Some factors associated with attrition include gender (males more than females) [19,23], adolescent substance use [23], poor physical health, coming from a family with a low-socioeconmic status, having parents who are divorced [19].

Several strategies to minimize attrition in longitudinal studies among adolescents such as sending of reminder letters to participants, repeat visits and use of incentives have been reported [24–28]. Other strategies include collection of detailed contact information about participants at baseline [28], collection of data from adolescents in school, repeat visits [28], use of postal questionnaires, telephone, or home interviews [16,23,28]. Attrition, however, remains a problem in longitudinal studies among adolescents. In addition, many retention strategies are resource intensive, and this is a challenge for longitudinal research in low- and middle-income countries [22].

Longitudinal studies among adolescents would be particularly beneficial in low- and middle-income countries such as Nigeria where adolescents comprise a significant proportion (23%) of the population [29]. Not only is there a dearth of longitudinal studies in Nigeria, but there is a dearth of funding which makes follow up challenging. Many of the follow-up methods are also somewhat difficult to implement in developing countries like Nigerian. For example, although there has been a general increase in access to mobile phones and internet access, many younger adolescents do not have personal phones [30] and use a parent or an older sibling or friend’s phone or computer for mobile phone and internet access. This limits the use of email reminders and retention strategies dependent on personal mobile phone ownership. Use of postal questionnaire and mailed reminder letters are also challenging because very few people receive mail at home through door-to-door delivery of mail. The majority collect their mail from a personal mailbox within the post office. This however requires an annual subscription. Currently, literacy rates among adolescents and their parents are also not universal and this precludes the use of strategies that require participants to be literate.

In view of the documented benefits of longitudinal studies among adolescents, we conducted a pilot longitudinal study among adolescents selected from the first year in junior secondary schools in Ibadan, Nigeria to (i) determine changes in the prevalence of selected behaviours (sexual practices, psychoactive substance use) and health conditions (including intentional and unintentional injuries) over time and (ii) determine the feasibility of long-term follow-up of the in-school adolescents using affordable retention strategies applicable within our context. Our study focused on adolescents in the first year in secondary school for various reasons. First, they are likely to be in their early adolescent years and this is a time of rapid bio-psycho-social changes which impact on their physical, mental, and emotional health. Although risk-behaviours typically commence during the later adolescent years compared to the early years, some younger adolescents do engage in risky behaviours [29,31,32]. Furthermore, the early adolescent years provide opportunity to promote positive behaviours and implement prevention interventions before initiation of health risk behaviours [31]. Finally, focusing on early adolescents provided opportunity for us to identify additional factors that could promote or hinder healthy development during the early adolescent years which may be missed if this group remains excluded from research. For this pilot, we limited our sampling to in-school adolescents because selection of samples from schools has been shown to promote retention among adolescents in longitudinal studies [28]. Epstein and colleagues further noted that for in-school adolescents, dropout rates are relatively low when data collection commences in the first year of middle or junior high school because students generally remain in the school for two more years except when the family relocates [28]. This strategy is thus suited to the Nigerian context where secondary education spans six years – three years of junior secondary and three years of senior secondary schooling. In the current paper, we present the findings on willingness to participate in longitudinal studies and factors associated with attrition among young in-school adolescents in Ibadan, Nigeria. Findings provide information on priority strategies to reduce attrition so that scarce resources can be allocate efficiently.

## Materials and methods

In this longitudinal study, in-school adolescents were recruited from selected secondary schools in Ibadan North Local Government Area (LGA), Oyo state, Nigeria using multi-stage sampling. Ibadan North was purposively selected because it is the host community for the in-country investigators’ institution and networks with schools and adolescent groups in the LGA exist.

Our sample size calculation was conducted using the minimum sample size estimates for cross-sectional studies, $\text{N = }\frac{\text{π(1}-\text{π)}}{{\text{e}}^{\text{2}}}$ [33]

Where,

N =  estimated minimum sample size

π = proportion of adolescents exhibiting the attribute being investigated

e = Required size of standard error = $\frac{\text{precision }\left(\text{d}\right)}{{\text{Z}}_{\text{α}}}$

Z_α_ = standard normal deviate corresponding to the probability of making a type I error (α), 0.05 (5%)

 = 1.96

d = 0.05


$$\frac{\text{N}= \pi 1– \pi \text{x}{{\text{Z}}_{\alpha }}^{2}}{{\text{d}}^{2}}$$

The minimum sample size was calculated based on the objectives of the main study which were to examine changes in selected health-risk behaviours. Thus, the proportions of adolescents who were current consumers of alcohol (12.4%) [32] current cigarette smokers (3.5%) [32] or who initiated sex before 16 years as a proxy for unsafe sex (9%) [34] were used to calculate the minimum sample size. The largest sample size calculated – 260.8 was adjusted for clustering in the sampling by multiplying this sample by 2, giving 522. An additional 20 percent of 522 was added to account for attrition, giving a minimum sample size of 626. A total of 1067 adolescents were eventually recruited. Details of the sample size calculation are in S1 File.

Using a table of random numbers, 10 government-owned and 10 private secondary schools were selected from the list of 52 private and 36 government-owned schools within the LGA. The school heads of two of the private schools initially selected declined to participate. These schools were replaced with the next school on the list of schools within the study area. In each school, up to 50 students were selected from the first year in junior secondary school to allow us to focus on changes in risk behaviours during the early adolescent years. We anticipated that there would be minor loss to follow up as we could avoid the transition from junior to senior secondary school. In government and private schools with more than one first year class, we continued to include additional classes sufficient to meet our sample size. In most instances one to two first year classes were selected). Individual students were then selected by simple random sampling after stratification by gender. In private schools with only one first year class, that class was chosen, and all eligible students were selected. Eligible participants were adolescents in the first year in secondary school who were willing to participate in the study, and from whom parental consent and adolescent assent were obtained.

The study instrument, a questionnaire, was used to obtain information on socio-demographic and family characteristics, family support, peer and school connectedness, selected health-risk behaviours (including sexual practices, cigarette smoking, drinking of alcohol), and health conditions (injuries, general health complaints). Questions were drawn from the Health Behaviour in School-age children Survey [35,36] and the Youth Risk Behaviour Survey questionnaires [37]. Data was collected over three data collection waves, one year apart. As a result of delays in obtaining approval from some schools and replacing schools that declined to participate and the onset of holidays, data collection eventually took place between April and September 2017 (wave one); June and October 2018 (wave two), and August and November 2019 (wave three). A unique identification number was assigned to each student at the start of data collection. The identification numbers, mobile phone numbers and house addresses or descriptions of the location of the home where no formal addresses existed were entered and stored securely and accessible only to investigators and research staff.

At each wave, trained research staff administered the questionnaire to the students in a quiet space within the school premises where privacy was assured. During follow-up data collection, one of the investigators and/ or a research staff who had collected data in the school during the preceding wave first visited the schools to remind principals and students about the study and upcoming data collection. Research staff subsequently visited the schools to interview the adolescents at the agreed time. Research staff returned to the schools up to two times to ensure that all available students were interviewed. Principals, class teachers and other students provided information that students not found in the schools could have moved to other schools, relocated with their parents, or dropped out. They also provided the names of possible schools these students could have relocated too. Research staff visited the schools and were able to interview some adolescents (about 12). Two of the private schools closed permanently between waves two and three, and research staff were unable to track students from these schools. These students formed part of those lost to follow up at wave three. Attempts were made to reach all students who were not found in school by mobile phone calls (up to three separate occasions) and home visits (at least one visit attempted). These students could however not be traced as the mobile phone numbers provided did not go through, or calls were picked by neighbours or acquaintances who were reluctant to provide information about the whereabouts of the adolescent. Attempts to trace them using their home addresses were not successful.

**Retention strategies employed were:**

i. Recruiting adolescents in their first year in junior secondary school and involvement of school principals in recruitment
ii. Keeping of a participant register and obtaining information on the home addresses of the adolescents to facilitate home interviewing if adolescents were not found in school
iii. Obtaining a mobile phone number provided by the adolescent (this was the adolescent’s number or that of a preferred parent/guardian) to enable the research team to track adolescents
iv. Repeat visits to schools to track missing students
v. Provision of small incentives (a pen and exercise books) to the adolescents in appreciation of the time they spent filling out the questionnaire

### Data analysis

Descriptive analyses (frequencies and means) and logistic regression were conducted.

**Study measures**

i. Sociodemographic and family characteristics of the adolescents:
  School type (private or government-owned); gender (male or female); age at last birthday; living arrangement (whether participant currently lived with one or both parents or a guardian); ownership of a mobile phone (yes or no); currently working for remuneration (yes or no); Family characteristics: type or marriage (monogamous or polygamous); father and mothers’ employment status
ii. Willingness to participate in a longitudinal study: proportion of students approached who stated that they would be willing to participate
iii. Acceptability of selected follow-up strategies: proportion of students who indicated that they were willing to be followed up via home visits, phone calls, SMS and social media-based messaging.
iv. Attrition
  Attrition at data collection wave two: Proportion of adolescents not interviewed at wave two compared to sample at baseline
  Attrition at data collection wave three: Proportion of adolescents not interviewed at wave three compared to sample in wave two
  Overall attrition: Proportion of adolescents who could not be tracked and interviewed at waves two and three compared to the total sample at baseline (wave one)
v. **C**auses of attrition during data collection waves two and three: Proportion of all students lost to follow up for each of the reasons identified.

### Factors associated with attrition

Binary logistic regression analysis was conducted to determine baseline socio-demographic characteristics associated with overall attrition over the entire data collection period. Adjusted odds ratio and 95% confidence interval are reported.

### Ethics approval

Ethical approval was obtained from the Oyo State Ethical Review Committee (Reference number: AD 13/1479/467). Permission to conduct the study was also obtained from the Ministry of Education and the school principals. The purpose of the study was explained to the school principals, and they gave permission to access the school after reading through the questionnaire. The principal, parents and adolescents were informed that the research was to assess the occurrence of selected behaviours and health conditions over time, and students would be interviewed up to three times. In addition, the researchers were also interested in understanding the possibility of enrolling and following the adolescents in research conducted over a period of time. Parents provided written consent (school principals assisted with contacting parents) and adolescents provided verbal assent. Each adolescent was taken through the informed assent process by the interviewer who administered the questionnaire. The field supervisor was the witness. Adolescents were assured that participation was confidential, voluntary, they did not have to answer every question and could chose to end the interview at any time, and they would not suffer any consequences if they chose not to participate or complete the interview.

## Results

### Adolescent socio-demographics characteristics

A total of 1067 adolescents were interviewed at wave one, and 673 and 358 in the second and third waves respectively; 539 adolescents were interviewed from government-owned and 528 from private schools. Their mean age at baseline was 11.9 ±  1.2 years, 871 (81.6%) students were currently living with both parents and 34.9% owned a personal mobile phone; 83.1% were from monogamous families, and parents of more than 90.0% of the students were currently employed (Table 1).

**Table 1:** Socio-demographic and family characteristics of the adolescents at baseline.

Socio-demographic and family characteristics		N	%
**Gender (n = 1067)**	Male	533	50.0
	Female	534	50.0
**Living arrangement (n = 1067)**	Both parents	871	81.6
	One parent	128	12.0
	Guardian	68	6.4
**Ownership of personal mobile phone (1053)**	Yes	367	34.9
	No	686	65.1
**Currently working for remuneration (n = 1059)**	Yes	99	9.3
	No	960	90.7
**Family type (n = 1059)**	Monogamous	880	83.1
	Polygamous	179	16.9
**Father’s employment status (n = 1055)**	Employed	1014	96.1
	Not employed	18	1.7
	Don’t know	23	2.2
**Mother’s employment status (n = 1054)**	Employed	1001	95.0
	Not employed	45	4.3
	Don’t know	8	0.8

### Willingness to participate in a longitudinal study

The majority 1067 (99.4%) of all 1073 adolescents approached agreed to be part of this pilot and were eligible. Of the 1067 adolescents, 875 (82.0%) stated that it would be acceptable if they were followed up by home visit, phone call, text message or chat-based messages. Three hundred and eighteen (approximately 29 percent) declined home visits as a means of follow-up. Some reasons for declining were as follows: adolescent not certain if parent/ guardian would permit a home visit, 36 (11.8%), adolescent did not want a home visit, 31 (10.1%) adolescent and family members were often not available at home 27 (8.8%). More than a third 384 (36.0%) were willing to be followed up using mobile phone-based strategies, 21 of whom did not have personal phones but who could be reached through a parent. About a fifth declined the use of phone calls because they could not remember their phone number, while 11.1% declined because they required parental permission to use their phones or give out their numbers. (Table 2).

**Table 2:** Acceptability of the study retention strategies.

Acceptability of study retention strategies		N	(%)
Home visits (n = 1067)	Yes	749	70.2
	No	318	29.8
Reasons for declining home visits (n = 306)			
	Not certain if parent/guardian would permit home visit	36	11.8
	Adolescent does not want a visit	31	10.1
	Adolescent and/or family members not often at home	27	8.8
	Research team members not known to family/ strangers/security reasons	20	6.5
	Home address unknown/ not easy to locate	17	5.6
	Parental permission required	15	4.9
	Home is far	13	4.2
	Parent/ guardian would be upset	10	3.4
	Family/ adolescent does not welcome visitors	9	2.9
	House not good enough	4	1.3
	Others^a^	20	6.5
	No specific reason/ no response	104	34.0
Phone ownership (n = 1053)	Yes	367	34.9
	No	686	65.1
Mobile phone-based strategies (n = 1067)	Yes	384	36.0
	No	683	64.0
Acceptability of specific mobile-phone based follow-up strategies	Phone call (n = 1067)	227	21.3
(Multiple response)	SMS (n = 1067)	153	14.3
	Social media^b^ (n = 1067)	51	4.8
Reasons for declining phone calls to personal number (n = 99)	Phone number not known/forgotten	21	21.2
	Parental instruction not to use phone/ insert a SIM into phone or give out number	8	8.1
	Parental permission required	3	3.0
	Research team unknown to participant/ family	2	2.0
	No specific reason	35	35.4
	Others^c^	30	30.2

^a^Others – adolescent always busy, adolescent/ family desire privacy, adolescent resides in the boarding house.

^b^WhatsApp.

^c^Others - has no phone, no sim card, parents hold on to phone, doesn’t want to give researchers number etc., doesn’t make calls on phone

### Attrition and reasons for attrition

**Fig 1:**
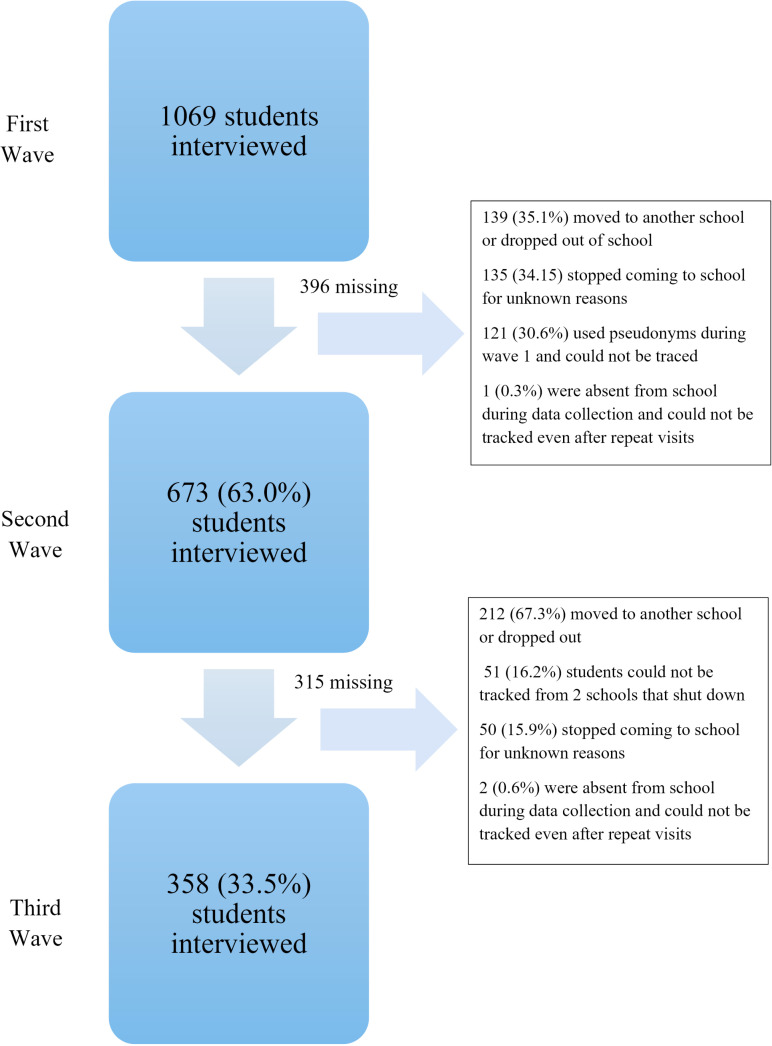
Reasons for attrition among the adolescents.

During data collection wave two, 671 of the 1067 adolescents re-interviewed while 396 were lost to follow-up, giving an attrition rate of 37.1%. Reasons for attrition of these 396 adolescents were as follows: 139 (35.1%) were said to have either relocated to a different school or dropped out, 135 (34.1%) had stopped coming to school for unknown reasons and 121 (30.6%) had used a pseudonym during the first data collection wave, which was unknown to research staff at that time, and thus they could not be traced in their schools (Fig 1). Some students who used pseudonyms introduced themselves to the data collection staff but could not remember the pseudonym used or their study identification number. It was possible to link some of those using pseudonyms using previously collected socio-demographic information. However, for 85 of them, although wave two data was collected, it was not possible to link their wave two data with their wave one data.

During wave three data collection, 358 (53.2%) of the 671 adolescents interviewed in wave two were interviewed; 315 were lost to follow-up between waves two and three, giving an attrition rate of 46.9%. The main reasons for attrition of these 315 adolescents were: change of school or dropout 212 (67.3%) and closure of two private schools and inability to trace 51(16.2%) students recruited from these schools.

In all, 711 (396 between waves one and two and 315 between waves two and three) of the 1067 adolescents recruited at baseline were lost to follow-up giving an overall attrition rate of 66.5% (95% CI =  63.67 – 69.33). Of the 358 adolescents interviewed in wave three, 289 had data for all three data collection waves which could be linked, 35 had data for all three waves but waves two and three data could not be linked to wave one data because they had used pseudonyms in wave one. Thirty-four adolescents were interviewed in waves one and three but not wave two because they had been absent from school during wave two. All adolescents interviewed in waves two and three were tracked through their schools. We were unable to locate any of the adolescents not found in school using their home address or mobile phone-based follow-up.

### Characteristics of adolescents ever and never lost to follow-up

Baseline socio-demographic characteristics of adolescents ever lost to follow-up (and those whose data could not be linked across all three study waves), and those never lost to follow-up were largely comparable. However, 55.9% of all those ever lost to follow, up, were enrolled in private schools compared with 32.2% of those not lost to follow-up. Parents of 84.7% adolescents lost to follow-up were in monogamous marriages compared to 78.7% of those not lost to follow -up (Table 3).

**Table 3:** Baseline socio-demographic and family characteristics of the adolescents by attrition status.

		Ever lost to follow-up^a^		
**Socio-demographic and family characteristics**		**Yes** **n = 778**	**No** **n = 289**	**p-value**
School type (N = 1067)	Private	435 (55.9)	93 (32.2)	
	Government-owned	343 (44.1)	196 (67.8)	<0.001*
Gender (N = 1067)	Male	384 (49.4)	149 (51.6)	
	Female	394 (50.6)	140 (48.4)	0.523
Age (N = 1064)	Mean =	11.9 ± 1.2	12.1 ± 1.2	0.179
Living arrangement (N = 1067)	Both parents	627 (80.8)	242 (83.6)	
	One parent	103 (13.3)	25 (8.7)	
	Guardian	46 (5.9)	22 (7.7)	0.087
Ownership of personal mobile phone (N = 1055)	Yes	268 (35.0)	99 (34.4)	
	No	497 (65.0)	189 (65.6)	0.885
Currently working for remuneration (N = 1059)	Yes	79 (10.2)	20 (7.0)	
	No	696 (89.8)	264 (93.0)	0.123
Parents’ type of marriagea (N = 1059)	Monogamous	654 (84.7)	226 (78.7)	
	Polygamous	118 (15.3)	61 (21.3)	0.027*
Father’s employment status (N = 1055)	Employed	733 (95.4)	281 (97.9)	
	Not employed	17 (2.2)	1 (0.3)	
	Don’t know	18 (2.4)	5 (1.7)	0.074
Mother’s employment status (N = 1054)	Employed	734 (95.7)	267 (93.0)	
	Not employed	27 (3.5)	18 (6.3)	
	Don’t know	6 (0.8)	2 (0.7)	0.139

*p < 0.05.

^a^Participants classified as lost to follow-up included those who could not be interviewed during either or both waves 2 and 3, and those whose wave 1 data could not be linked to their subsequent data because they could not remember the pseudonym, they used in wave 1.

^b^Monogamous marriage in which the father is married to one wife while in a polygamous marriage, the father is married to more than one wife

### Factors associated with attrition

Data from 289 of the 358 students interviewed in wave three could be linked across all the three data collection waves. These 289 were categorized as never lost to follow-up and used for the analysis of factors associated with attrition. Adolescents attending private schools were 3.4 times more likely to have been lost to follow-up compared to adolescents attending government-owned schools. Adolescents who did not own a personal mobile phone were 1.4 times more likely to have been lost to follow-up compared with those who had no personal mobile phone. Furthermore, adolescents who reported engaging in some work for remuneration at baseline were about twice as likely to be lost to follow-up compared to their counterparts who were not engaging in work for remuneration (Table 4).

**Table 4:** Factors associated with attrition over the entire study period.

		Ever lost to follow-up			
**Variable**		**Yes** ^a^ **n = 778**	**No** **n = 289**	**AOR**	**95% C.I.**
School type	Private	435 (82.4)	93 (17.6)	3.35	2.39 – 4.69*
	Government-owned	343 (63.6)	196 (36.4)	1	
Gender	Male	384 (72.0)	149 (28.0)	1	
	Female	394 (73.8)	140 (26.2)	1.19	0.89 – 1.59
Age Mean (SD)	–	11.9 (1.2)	12.1 (1.2)	1.09^b^	0.96 – 1.24
Ownership of a mobile phone n = 1053)	Yes	268 (73.0)	99 (27.0)	1	
	No	497 (72.4)	189 (27.6)	1.43	1.03 – 1.98*
Currently working for remuneration (N = 1059)	Yes	79 (79.8)	20 (20.2)	2.04	1.19 - 3.49*
	No	696 (72.5)	264 (27.5)	1	
Living arrangement	At least one parent	104 (80.5)	25 (19.5)	1.74	0.84 – 3.60
	Both parents	627 (72.4)	239 (27.6)	0.95	0.53 – 1.69
	Guardian	46 (67.6)	22 (32.4)	1	
Family type (n = 1061)	Monogamous	654 (74.3)	226 (25.7)	1.46	0.99 – 2.16
	Polygamous	118 (65.9)	61 (34.1)	1	
Father’s employment status (N = 1055)	Employed	733 (72.3)	281 (27.7)	0.84	0.28 – 2.50
	Not employed	17 (94.4%	1 (5.6)	6.87	0.69 – 68.93
	Don’t know	18 (78.3)	5 (21.7)	1	
Mother’s employment status (N = 1054)	Employed	734 (73.3)	267 (26.7)	1.81	0.93 – 3.50
	Not employed	27 (60.0)	18 (40.0)	1	
	Don’t know	6 (75.0)	2 (25.0)	1.320	0.21 – 8.27

*p < 0.05.

^a^Participants included those who could not be interviewed during either or both waves two and three and those interviewed in wave two, but whose wave one data could not be linked to their subsequent data because they could not remember the pseudonym, they used in wave one.

^b^Mean difference

## Discussion

We assessed willingness to participate in longitudinal studies and factors associated with attrition among in-school adolescents in Ibadan, Nigeria. The majority of the adolescents were willing to participate in this study and this is similar to findings from other studies that reported high levels of willingness to participate in research amongst adolescents [15,38,39]. While more than 80% of the adolescents were willing to be followed up using at least one retention strategy, approximately 30% did not want to be followed up via home visits. This was mainly because they wanted parental approval for home visits first and were not familiar with the research team. Other researchers have emphasized the importance of engaging and building a relationship with parents, guardians, and other important stakeholders to promote participation and retention of adolescents in longitudinal studies [40,41] Almost 40% of the adolescents in our study were willing to be followed up using a mobile phone-based strategy, but few of them owned personal mobile phones. Reports from our study area indicate that younger adolescents are less likely to own personal mobile phones compared to older adolescents [30]. Engaging with parents/ caregivers could also overcome this barrier as adolescents can be reached using their parents/ caregivers’ mobile phones.

Our overall attrition rate (67%) though high, is within the range of 25% to as high as 75% reported in various adolescent longitudinal studies [17–21]. One of the reasons for attrition in the current study was the use of pseudonyms by some students. Unknown to research staff, some adolescents used a pseudonym (different from their formal names in the class register) and this was discovered during wave two. Information collected in subsequent waves could therefore not be linked to their baseline information as many forgot the pseudonyms used. Name discrepancies among participants because of differences in spellings or participants using native as opposed to English names were also reported in the Birth-to-twenty study [42]. Although the reasons for the name discrepancies were different from our experience, the effects, namely, difficulty following up participants, and linking their data are similar [43]. Our adolescents could have opted for pseudonyms for various reasons. First, the fact that interviews were conducted in schools could have raised concerns about the anonymity of their responses. This concern has been noted in school-based research among adolescents [28]. Secondly, although none of the students explicitly stated that they did not trust the researchers, some of their reasons for declining home visits, i.e., research team members are not known to family, research team members are strangers, suggested that they might have viewed the researchers as strangers and were thus wary about divulging some information. It is important to explore additional reasons for the use of pseudonyms by adolescents in our setting because of its effect on data completeness and linkage.

Other reasons for loss to follow-up in our study were relocation of family to a different neighbourhood or state, change of school and closure of some schools. These reasons have also been documented by other researchers [28,44]. Attrition was further affected by the timing of wave three data collection. This took place during the first term of a new session after students had been promoted from junior secondary class three to senior secondary class one. Thus, a considerable number of the adolescents had either moved to different senior secondary schools having completed three years of junior secondary education in the recruitment schools or dropped out of school altogether [45]. Epstein et al (2000) noted that the likelihood of attrition is higher among in-school adolescents in transitional classes - near the end of middle school or end of secondary school because they are more likely to move to other schools or move with their families compared with those in the early years in these classes who are more likely to remain in the school until completion of middle or high school [28,46]. The high prevalence of school dropout between junior and senior secondary school has also been documented in the study area - Oyo state [45].

The baseline socio-demographic characteristics of adolescents lost to follow-up and those not lost to follow-up were largely comparable. Findings from other studies are varied and some find that those lost and not lost to follow-up are generally comparable [20], whereas others find notable differences in adolescents lost and those not lost to follow-up [47]. We further found that among students lost to follow-up, 79% were from families in which the parents were in a monogamous marriage, i.e., the father has only one wife compared to 85% of those from families where the father had more than one wife. This finding was not statistically significant on logistic regression, and possibly occurred because parents in monogamous marriages might be better able to afford private schooling for their children because of smaller overall family sizes compared to those in polygamous marriages. The significantly higher attrition among adolescents attending private schools might thus have been responsible for this observation.

In the current study, baseline socio-demographic factors associated with attrition were attending a private school, not owning a personal mobile phone and engaging in remunerated work. These were different from characteristics such as adolescents’ age, gender, family characteristics and substance use reported by other studies [18,21,23,47,48]. Lower interest in the study among adolescents in private schools could also have contributed to the higher attrition rate. Richter et al (2004) had documented that families that were better-off were reluctant to participate in the Birth-to-twenty longitudinal study [12]. Differences in interest in participation by family income level would need to be explored in our setting. An important reason for the higher loss to follow-up among adolescents attending private schools that we found was because of an unexpected movement of students from private to government schools. This was because the political party elected in the state in 2019 (when wave three data was collected) implemented education policies in government schools such as cancellation of school fees, and provision of textbooks and exercise books to students. Many parents thus withdrew their children from private schools and enrolled them in government-owned schools. This contributed to the closure of two private schools where 137 of our students were recruited. Our finding that students who did not own a personal mobile phone were more likely to be lost to follow-up has implications for retention, because owning a personal mobile phone could make it easier to maintain contact between consecutive data collection waves. Use of phone calls is a documented strategy for enhancing retention and increasing availability of mobile phones made it easier to reach parents and children in the Birth-to-twenty longitudinal study in South Africa [49]. However, given that we were unable to track any students using the mobile phone numbers they provided, the association between mobile phone ownership and retention needs further enquiry. Working for remuneration (often driven by poor family economic circumstances) [50] is associated with school absenteeism and drop out [50–52]. and could explain why adolescents engaging in paid work were more likely to be lost to follow-up in the current study.

Of all the retention strategies adopted – recruitment of adolescents through schools which enabled repeat visits to schools was the only strategy through which students were located and interviewed in subsequent data collection waves. Recruiting adolescents through schools is widely recognized as a means of recruitment and retention in adolescent longitudinal studies [28,53]. School-based recruitment and follow-up could thus be an important strategy for promoting retention in school-based longitudinal studies in our setting. Bearing in mind that our attrition rates were high, instituting multiple resource-intensive strategies simultaneously [26,54,55] could be more useful for ensuring retention compared to the low-cost strategies we utilized. Some of these resource-intensive strategies include actively engaging parents to obtain buy-in and permission for home visits, use of mobile phone calls and social media to maintain contact with the adolescents; verifying tracking information, e.g., by conducting home visits and making phone calls immediately after obtaining parental buy-in, providing adolescents with a schedule card containing their identification number, and engaging and assigning a particular staff member to track adolescents from the beginning through to the end of the study. In addition, regular revision of the protocol to include newer retention strategies especially those that offer flexibility to participants (e.g., offering to do the interview using a video-based voice-over-internet platform) could improve retention among our study participants [26,54,55].

## Conclusion

Adolescents in Ibadan are willing to participate in a longitudinal study, however reliance on minimal low-cost retention strategies was associated with high attrition. Our pilot emphasizes that despite the additional expense, multiple, active, cost-intensive retention strategies are required to minimize attrition. This finding is important as funding agencies may question why cheaper methods (such as mobile phones) alone, compared to time and resource intensive home visits are not used. Formative research may also be required to understand the adolescent population being targeted, reasons for use of pseudonyms by adolescents, and strategies that would work best among particular populations of adolescents.

### Limitations

We used an interviewer-administered mode of data collection, and some adolescents might have provided answers they felt would be more acceptable. To reduce this possibility, we engaged trained research staff with experience working and building a rapport with adolescent in research and non-research settings. The adolescents were also assured of confidentiality of responses. They were further reminded that they did not have to respond to questions if they did not feel like. Teachers and peers provided information that some students were not in school because they had either moved to other schools or dropped out, we were however unable to verify these reports among students lost to follow-up. The fact that this study focused on young adolescents, who largely rely on their parents for decision making could have contributed to adolescents not giving correct home addresses thus increasing attrition. The authoritarian parenting style which is common in our culture could also have influenced adolescents’ decision to use pseudonyms and withhold correct information about home addresses and mobile phone numbers. These issues need to be taken into consideration when conducting longitudinal studies with young adolescents in our environment. Despite this limitation, our study provides very useful information which would be useful in setting up longitudinal studies amongst adolescents in our setting and in settings comparable with ours.

## Supporting information

S1 File Sample size estimation. (DOCX)

S2 File Dataset for factors associated with attrition in a longitudinal study among adolescents_PLoSOne_2022. (XLSX)
