# Supplementary material for: Factors associated with attrition in a longitudinal study of health risk behaviours and conditions among adolescents in Ibadan, Nigeria
Source: PLoS One. 2025 Apr 4;20(4):e0320150. doi: 10.1371/journal.pone.0320150 (PMC11970656; doi:10.1371/journal.pone.0320150)
Supplement: S1 File — (DOCX) [file pone.0320150.s001.docx]

**Sample size estimation**

The following sample size formula was used

N = π (1 – π) [1]

e^2^

Where

N = estimated minimum sample size

π = proportion of adolescents exhibiting the attribute being investigated

e = Required size of standard error = precision (d)

Z_α_

Z_α_ = standard normal deviate corresponding to the probability of making a type I error (α), 0.05 (5%)

= 1.96

d = 0.05

N = π (1 – π) x Z_α_^2^

d^2^

π = Proportion of adolescents who are current users of alcohol or current cigarette smokers or currently sexually active (these were some of the risk behaviours studied

prevalence of current alcohol use among adolescents = 12.4% [2]

prevalence of current cigarette smoking among adolescents = 3.5% [2]

proportion of adolescents who had unsafe sex (sex before 16 years) = 9 % [3]

N = 0.124 x 0.876 (1.96)^2^

(0.05)^2^

= 260.8 x 2 (to account for clustering design in the sampling technique) = 522

= 522 + (20% to account for attrition)

= 522+104

= 626

*The largest estimated minimum sample size (626) was obtained using the prevalence of current alcohol consumption and this was used in this study.

A total of 1067 adolescents were eventually recruited at baseline

**References**

1. Kirkwood, Betty R and Sterne, Jonathan AC (2010), *Essential medical statistics* (John Wiley & Sons).
2. Olumide, Adesola O, et al. (2014), 'Predictors of substance use among vulnerable adolescents in five cities: findings from the well-being of adolescents in vulnerable environments study', *Journal of Adolescent Health,* 55 (6), S39-S47.
3. Fatusi, Adesegun and Blum, Robert (2008a), 'Predictors of early sexual initiation among a nationally representative sample of Nigerian adolescents', *BMC Public Health,* 8 (1), 136.
